# Deep Neural Network for Reducing the Screening Workload in Systematic Reviews for Clinical Guidelines: Algorithm Validation Study

**DOI:** 10.2196/22422

**Published:** 2020-12-30

**Authors:** Tomohide Yamada, Daisuke Yoneoka, Yuta Hiraike, Kimihiro Hino, Hiroyoshi Toyoshiba, Akira Shishido, Hisashi Noma, Nobuhiro Shojima, Toshimasa Yamauchi

**Affiliations:** 1 University Institute for Population Health King’s College London London United Kingdom; 2 Department of Diabetes and Metabolic Diseases Graduate School of Medicine University of Tokyo Tokyo Japan; 3 Graduate School of Public Health St Luke's International University Tokyo Japan; 4 Department of Cell Biology Harvard Medical School Boston, MA United States; 5 FRONTEO Healthcare Inc Tokyo Japan; 6 Department of Data Science The Institute of Statistical Mathematics Tokyo Japan

**Keywords:** machine learning, evidence-based medicine, systematic review, meta-analysis, clinical guideline, deep learning, neural network

## Abstract

**Background:**

Performing systematic reviews is a time-consuming and resource-intensive process.

**Objective:**

We investigated whether a machine learning system could perform systematic reviews more efficiently.

**Methods:**

All systematic reviews and meta-analyses of interventional randomized controlled trials cited in recent clinical guidelines from the American Diabetes Association, American College of Cardiology, American Heart Association (2 guidelines), and American Stroke Association were assessed. After reproducing the primary screening data set according to the published search strategy of each, we extracted correct articles (those actually reviewed) and incorrect articles (those not reviewed) from the data set. These 2 sets of articles were used to train a neural network–based artificial intelligence engine (Concept Encoder, Fronteo Inc). The primary endpoint was work saved over sampling at 95% recall (WSS@95%).

**Results:**

Among 145 candidate reviews of randomized controlled trials, 8 reviews fulfilled the inclusion criteria. For these 8 reviews, the machine learning system significantly reduced the literature screening workload by at least 6-fold versus that of manual screening based on WSS@95%. When machine learning was initiated using 2 correct articles that were randomly selected by a researcher, a 10-fold reduction in workload was achieved versus that of manual screening based on the WSS@95% value, with high sensitivity for eligible studies. The area under the receiver operating characteristic curve increased dramatically every time the algorithm learned a correct article.

**Conclusions:**

Concept Encoder achieved a 10-fold reduction of the screening workload for systematic review after learning from 2 randomly selected studies on the target topic. However, few meta-analyses of randomized controlled trials were included. Concept Encoder could facilitate the acquisition of evidence for clinical guidelines.

## Introduction

Evidence-based medicine aims to provide treatment that matches a patient’s needs by integrating the best and latest scientific evidence and clinical skills [1]. Performing systematic reviews and meta-analyses is vital to obtain data that can inform evidence-based clinical decisions as well as the development of clinical and public health guidelines [2].

When performing a systematic review, it is critical to minimize potential bias by identifying all relevant published articles through exhaustive and systematic screening of the literature, which can be an extremely time-consuming and resource-intensive process.

The Cochrane collaboration mandates reinvestigation and updating of published systematic reviews and meta-analyses every 2 years to maintain the novelty and quality of evidence [3], but this is an onerous task. As a single systematic review or meta-analysis usually requires 1 to 2 years to complete, only one-third of all Cochrane reviews are updated on time [4], and many reviews are obsolete or missing [5,6]. Therefore, the development of methods for the automation of the systematic review process has been suggested [7].

To reduce the time and cost of screening literature when performing systematic reviews, researchers have explored the use of active learning text classification systems to achieve semiautomated exclusion of irrelevant studies while retaining a high proportion of eligible studies for subsequent manual review [8,9]. However, little progress has been made for the following reasons. First, previous studies did not investigate well-characterized and high-quality data sets, so the type of systematic review used as the data source was unclear, and the method of applying machine learning to the clinical studies was obscure. Second, previous reports did not specify how active machine learning was used. Third, only an approximate 30%-50% reduction of the workload was achieved [8]. Fourth, a method that extracts 100% of the correct articles from the literature has not been developed because most studies use a targeted extraction of 95% as the primary outcome; despite the importance of not missing any eligible studies when performing systematic reviews (ie, the objective is to identify all relevant articles) [10-14].

To overcome some of these issues, we studied systematic reviews of randomized controlled trials cited in several recent international clinical guidelines to investigate whether an active machine learning system (Concept Encoder, Fronteo Inc) could reduce the workload and accelerate the review process while improving its precision.

## Methods

### Search Strategy and Selection of Reviews

This study was performed according to a specified protocol and was registered with the University Hospital Medical Information Network clinical trials registry (UMIN000032663). Our institutional review board waived the need for approval. Three reviewers (TYamada, HT, and NS) independently checked the reference lists of 5 recent clinical guidelines released by the American Diabetes Association [15], American College of Cardiology [16], American Heart Association (2 guidelines) [17,18], and American Stroke Association [19]. The reviewers identified all systematic reviews and meta-analyses cited in these guidelines with no language restrictions.

Next, the reviewers selected eligible systematic reviews and meta-analyses of interventional randomized controlled trials for medications that fulfilled the following inclusion criteria: First, a reproducible search strategy was required; therefore, articles with no description of the search strategy, or without a clear, reproducible description of the search strategy were excluded. In addition, meta-analyses using individual data, meta-analyses of observational studies, reports missing relevant information, and reviews of fewer than 5 studies were excluded. Finally, reviews were excluded if the primary screening data set did not include all of the correct articles (ie, those cited) when it was reproduced according to the published search strategy. Disagreements among the reviewers were resolved by consensus.

We reproduced primary screening data sets, including abstracts, according to reported search strategies, that is, a search strategy for PubMed was devised based on the search strategy for Ovid MEDLINE described in each review (Multimedia Appendix 1).

### Active Machine Learning System

An artificial intelligence engine (Concept Encoder) [20] was used to convert sentences into vectors, extract and learn each vector component as a feature value, identify similar vectors as indicators of the similarity of sentence content, and perform a rapid search for similar sentences. Vectorization facilitates text analysis by providing numerical data that allow various calculations to be performed (eg, to assess clustering of results). In addition, vectorization allows searches to be based on the sums and differences of sentences, facilitating comparison of content between 2 sentences and resulting in a sentence retrieval engine that can be adapted to research targets.

First, each sentence is decomposed into morphemes (the smallest meaningful units of a language) by morphological analysis, applying rules to label each morpheme level element with a word. Next, the word labels were embedded in the *k*-dimension vector space [21-24] using the word2vec technique. Sentences can also be embedded in the *k*-dimension vector space using an expansion to the word-embedding method called doc2vec that yields paragraph vectors [21-24]. Several parameters are used in these embedding techniques, such as the number of embedded words, the vectors' dimensions, and negative sampling (ie, the number noise samples, nonobserved data, generated in both word2vec and doc2vec algorithms). These algorithms enable the transformation into vectors of words and documents from articles in a systematic review. Assuming that there are a total of *m* abstracts and *n* words in all the articles (both reviewed and not reviewed) in a single systematic review or meta-analysis (ie, 1 of the 8 systematic reviews or meta-analyses included) embedded in a *k*-dimension vector space, then the abstracts and words can be expressed as

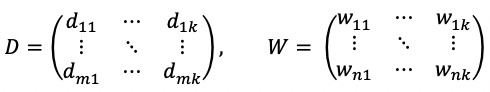



Embedded vectors are well known to possess interesting features such as word analogy and outperform the bag of word approaches in several linguistic tasks. For example, if 2 articles have similar contents, then the 2 row vectors in *D* associated with those articles are a short cosine distance from one another. Similarly, the 2 row vectors in *W* associated with 2 words having a similar meaning are also a short cosine distance from one another. Hence, if there are differences between the articles that were reviewed and not reviewed, then the reviewed articles should be closer to each other than those that were not reviewed. These features persist after the 2 matrices are multiplied due to linearity of multiplication. For example, if *w_i_* ≅ *w_j_* for 2 row vectors in matrix *W*, then the inner product with *d* (a row vector in matrix *D*) is *d* · *w_i_* ≅ *d* · *w_j_*. Expanding this to word analogy, if *w_i_* – *w_i_* ≅ *w_i'_* – *w_j'_* where *i*, *j*, *i'*, *j'* ∈ [1, 2, 3, ..., *n*] holds for 4 row vectors in matrix *W*, then *d* · *w_i_* – *d* · *w_j_* ≅ *d* · *w_i'_* – *d* · *w_j'_* is true for any row vector *d* in matrix *D*.

Hence, the product of these 2 matrices is a DW matrix, which is a sentence-word matrix that also possesses these interesting features of the original matrices.

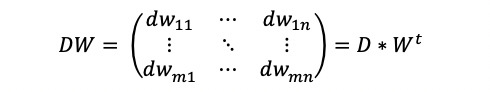



In this study, sentence similarity was evaluated by using a DW matrix.

Neural networks have previously been used to calculate *D* and *W* matrices, but calculation of these matrices becomes computationally intense when a large number of articles are investigated [21-24]. Hence, a neural network is generally restricted to embedding the 1000 most common words in *m* articles. In our analysis, the 1000 most common words were identified for each of the 8 studies.

A skip-gram model with negative sampling was chosen to calculate *W*. The embedding vector dimension was set at *k*=300, which is usually considered sufficient to capture word and document features, and the number of negative samplings was set at *n_s_*=5. A previous paper [25] reported that values of negative sampling in the range of *n_s_*=5-20 were useful for small training data sets, whereas for large data sets, *n_s_* can be as small as 2-5; the size of the data sets used in this study ranged from *m*=138 to *m*=6935.

For *D*, the distributed bag of words version of paragraph vector [20-24] was used as it is usually consistent across many tasks [24]. The same negative sampling and embedding dimension (*n_s_*=5 and *k*=300) were used. Both *D* and *W* were obtained at the same time in this study. However, it is possible to obtain *W* first and then calculate *D* by using the pretrained *W*. We used the gensim (version 3.8.3)[26] package for Python (version 3.6) with *n_s_*=5, *k*=300, and 1000 words.

A dimension reduction technique, such as singular value decomposition, can be used to approximate the DW matrix with a lower dimension matrix to reduce computational requirements; however, this was not done in this analysis (the number of columns in the DW matrix kept as 1000).

### Reproduction of the Reviews

The similarity of any 2 articles is defined as the cosine distance of the 2 vectors associated with these articles. After a correct (reviewed) or incorrect (not reviewed) article is identified, the associated row vector is defined as a correct or incorrect and used as the feature vector representing a correct or incorrect article. The cosine distances for all other articles (*m* − 1 articles) are calculated and arranged in descending order. For the next article from the top of the list, if the article is a correct one, the mean of the vectors for the correct articles is used to train Concept Encoder in the next step of active learning. If the article is an incorrect one, the vector is subtracted to train Concept Encoder in the next step of active learning, that is, it is used as the feature vector. Cosine distances between the updated vector and all other articles are calculated and ordered again, and this process is repeated until all of the correct articles have been identified. Here, the mean vector is simply used as the feature vector for the correct articles. We could build classification models using these vectors as features to arrange the remaining articles in a descending manner by active learning; however, similarity of articles seemed to be embedded in the vectors, and using the vectors directly as the features was effective. Therefore, we kept the process simple, and no further machine learning was conducted in our active learning process.

### Workload Reduction

Using 2 randomly selected correct articles (selected by Concept Encoder from among the correct articles), the following steps were performed to calculate how much workload reduction could be achieved using Concept Encoder.

**Figure 1:**
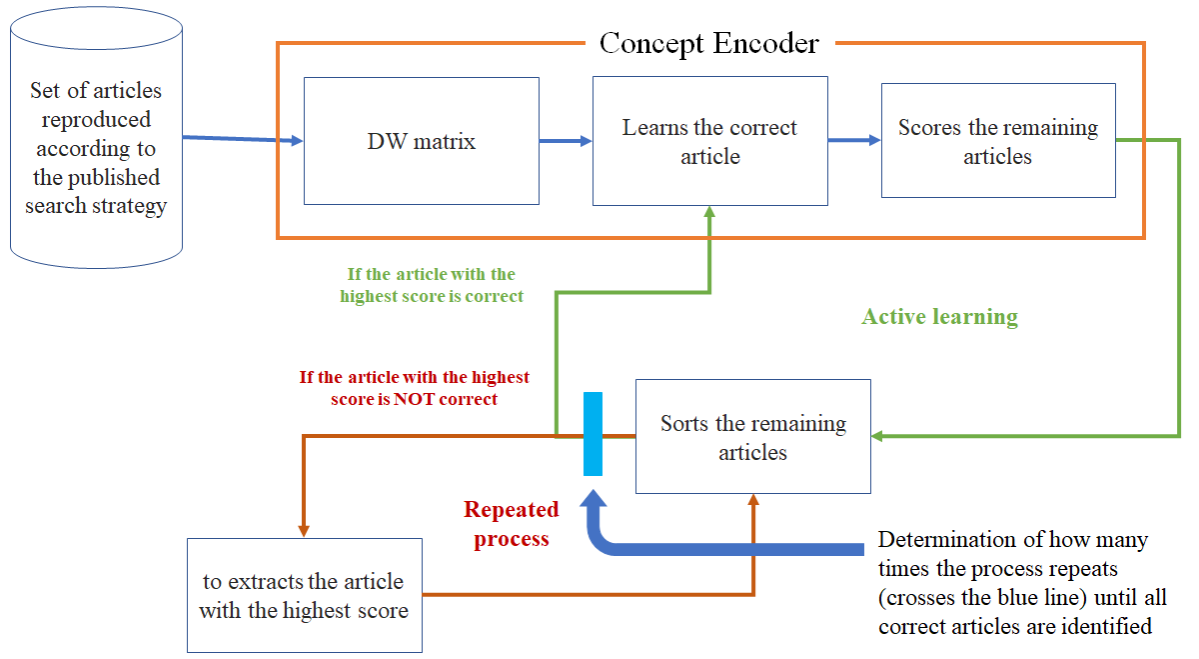
Flow diagram of information processing and user interaction with Concept Encoder.

1. Concept Encoder read these 2 articles and calculated the mean value of the sentence-word vectors corresponding to the 2 articles. Next, this mean value was used to assign scores to the other articles by determining the cosine distance between the mean value and the vectors corresponding to each of the remaining articles (Figure 1).
2. A researcher reviewed the article with the higher score. If this was a correct article, Concept Encoder learned it as a correct article based on the mean value of all chosen sentence-word vectors. If it was an incorrect article, the sentence-word vector is subtracted from the mean vector of the corrected articles.
3. Concept Encoder learned the correct and incorrect article, and thus identified and rescored the remaining articles, which had not been checked by the researcher.
4. The researcher again reviewed the article with the highest score. If this was a correct article, Concept Encoder learned it as a correct article. If it was incorrect, Concept Encoder learned it as an incorrect article.
5. After learning all of the correct and incorrect articles identified up to this point, Concept Encoder scored the remaining articles again. The mean of sentence-word vectors for all corrected articles minus the mean of sentence-word vectors for all incorrect articles was used to score the remaining articles.
6. Steps 2 to 5 were repeated until all of the correct articles had been identified. Following this, the final reading ratio was calculated as the number of articles read by Concept Encoder relative to the total number of articles. For example, if the total data set comprised 1000 articles, and Concept Encoder found all of the correct articles after reading 200 articles, the final reading ratio would be 20%, and the work involved in screening the literature would have been reduced by 80% (avoiding the need to read 800 out of 1000 articles). Work saved over sampling (WSS) @*R*% is an index to measure how much work is saved compared to manual screening to achieve identification of *R*% of correct papers.
7. Next, the first correct article (step 2) was changed, and the same process was repeated until all of the correct articles were identified.
8. The maximum reduction of the literature screening workload achieved by teaching Concept Encoder 2 correct articles (ie, 2 articles that were actually reviewed) was determined.

### Endpoints

The primary endpoint of this study was the reduction in the literature screening workload when Concept Encoder was used to identify all of the correct articles, relative to the workload for finding all of the correct articles by manual review with random sampling. WSS@95% recall was used for comparability as this endpoint is often used in previous studies (Multimedia Appendix 1).

### Statistical Analysis

WSS and receiver operating characteristics were used to evaluate the performance of the algorithm. Area under the receiver operating characteristic curve (AUROC) shows how much the active learning improves classification ability between correct and incorrect articles at each step of learning .

To evaluate the impact of the 2 initial papers selected on system performance, all possible pairs of papers were generated and used to run the algorithm. Then the mean and standard deviation of WSS@95% were measured. The confidence interval of the AUROC was determined at each step of the active learning process for all 8 studies using scores calculated from the cosine distances for articles that were used or not used in the systematic reviews.

## Results

**Figure 2:**
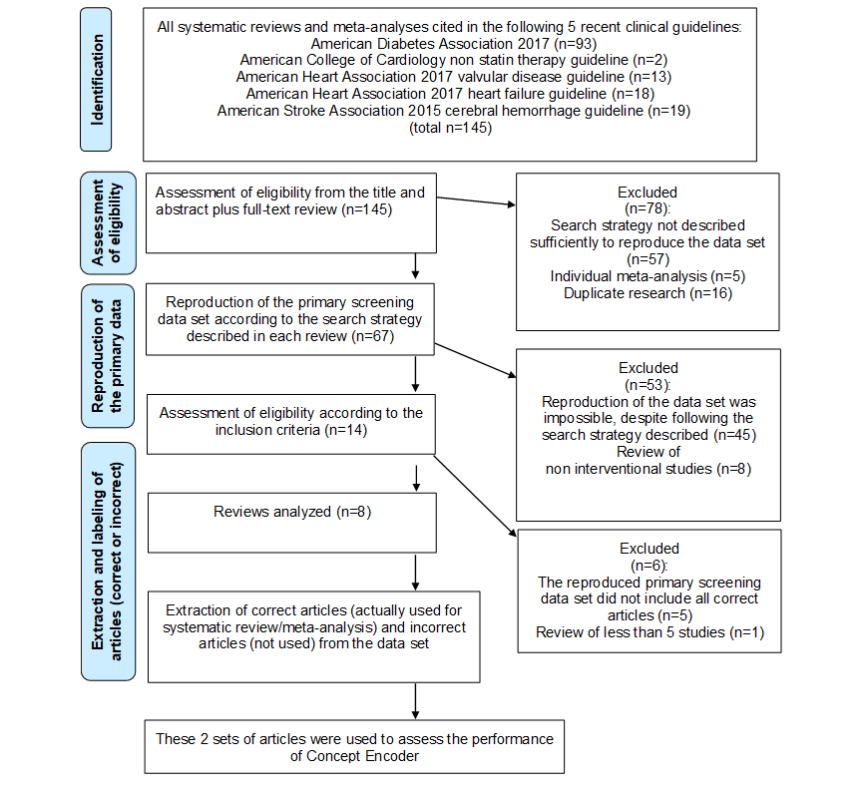
Literature search and study selection strategy.

A flowchart of our strategy for performing the literature search and study selection is shown in Figure 2. The systematic reviews and meta-analyses used in this study were cited in 5 recent clinical guidelines (93 from American Diabetes Association 2017 guidelines [15], 2 from American College of Cardiology guidelines for nonstatin therapy [16], 13 from American Heart Association 2017 guidelines for valvular disease [17], 18 from American Heart Association 2017 guidelines for heart failure [18], 19 from American Stroke Association 2015 guidelines [19]). Among the 145 candidate reviews, 137 were excluded, with the main reasons being that the search strategy was not described in sufficient detail to reproduce the data set (57 reviews), or the data set could not be reproduced despite following the described search strategy (45 reviews). A final 8 reviews published between 2012 and 2016 were selected [27-34]. These reviews comprised 2 Cochrane Database Systematic Reviews and 1 each published in JAMA Neurology, the British Medical Journal, PLOS Medicine, the Journal of the American Medical Association, the Lancet, and the Archives of Internal Medicine. The characteristics of these reviews are summarized in Multimedia Appendix 1.

After reproducing the primary screening data set (including abstracts) according to the search strategy described in each review, 81 sets of correct articles and 22,664 sets of incorrect articles were obtained (Multimedia Appendix 1). The search strategies employed for the reproduction of the data sets are detailed in Multimedia Appendix 1.

**Figure 3:**
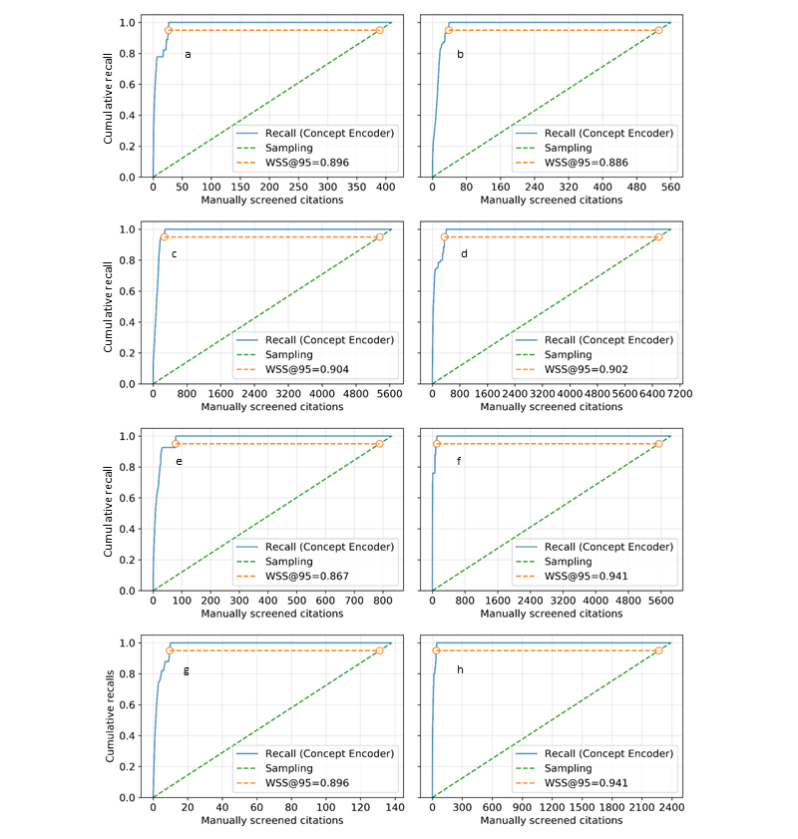
Average cumulative recall curves for all data sets: (a) Chatterjee et al [27], (b) Balsells et al [28], (c) Muduliar et al [29], (d) Yanovski and Yanovski [30], (e) Eng et al [31], (f) McBrien et al [32], (g) Andrade Castetllanos et al [33], and (h) Arguedas et al [34]. WSS: work saved over sampling.

One of the 8 studies contained only 140 articles. The number of words appearing more than twice in the data set was approximately 1200, including the stopwords. We also wished to examine the difference in performance between studies. Figure 3 displays the average cumulative recall curves for the 8 reviews. The performance of Concept Encoder was evaluated for every possible pair of articles chosen at the start of active learning. Concept Encoder was found to significantly reduce the workload by at least 0.867 compared with manual screening (the lowest mean WSS@95%). The average reduction of the workload compared with manual screening was >90% or 10-fold (WSS@95%: mean 0.904), and Concept Encoder showed a high ability to discriminate between correct and incorrect studies (Table 1). The choice of the initial 2 articles only had a small influence on the performance of the learning algorithm.

**Figure 4:**
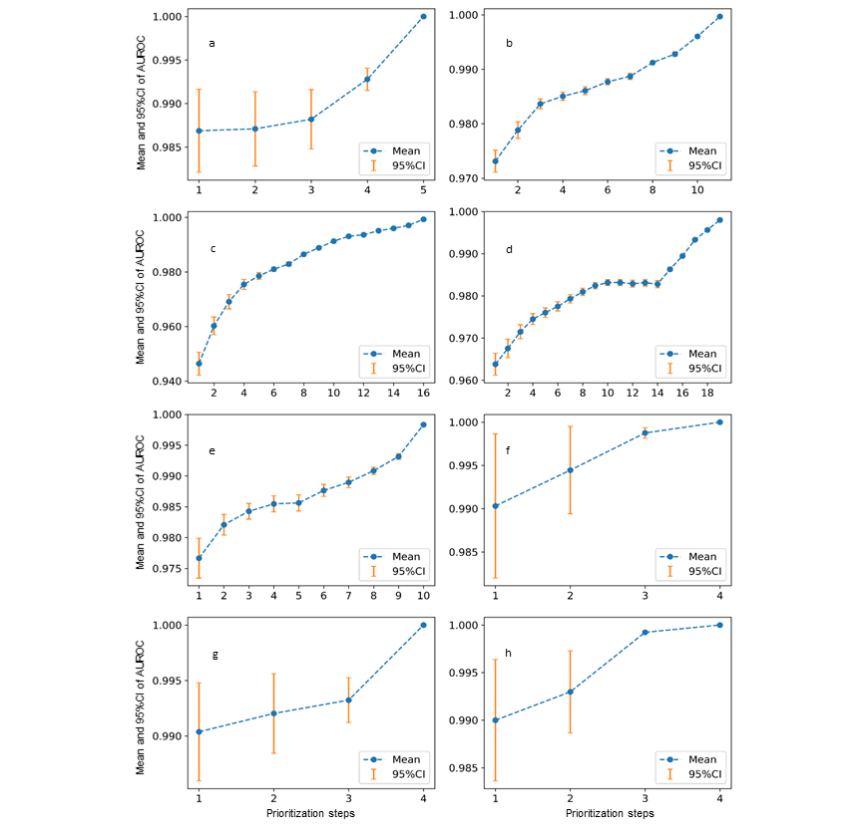
Performance for an increasing number of prioritization steps: (a) Chatterjee et al [27], (b) Balsells et al [28], (c) Muduliar et al [29], (d) Yanovski and Yanovski [30], (e) Eng et al [31], (f) McBrien et al [32], (g) Andrade Castetllanos et al [33], and (h) Arguedas et al [34]. AUROC: area under the receiver operating characteristic curve.

Prioritization (ie, the score based on cosine distance) of the algorithm by machine learning increased the AUROC to between 0.99 to 1.00, while the standard deviation of the AUROC decreased with each prioritization step (Figure 4).

**Table 1:** Review data sets and corresponding results.

Review				Concept Encoder					
Reference	Correct articles, n	Articles screened, n	Trials, n		AUROC^a^	WSS@100^b^		WSS@95^c^	
						Mean (SD)	Range	Mean (SD)	Range
[27]	6	410	15		1	0.946 (0.014)	0.937-0.985	0.896 (0.014)	0.887-0.935
[28]	12	560	66		1	0.936 (0.009)	0.930-0.959	0.886 (0.009)	0.880-0.909
[29]	17	5644	136		0.999	0.954 (0.006)	0.946-0.971	0.904 (0.006)	0.896-0.921
[30]	20	6935	190		0.998	0.944 (0.005)	0.941-0.957	0.902 (0.006)	0.897-0.932
[31]	11	830	55		0.998	0.917 (0.023)	0.906-0.975	0.867 (0.023)	0.856-0.925
[32]	5	5839	10		1	0.991 (0.006)	0.981-0.999	0.941 (0.006)	0.931-0.949
[33]	5	138	10		1	0.946 (0.015)	0.935-0.978	0.896 (0.015)	0.885-0.928
[34]	5	2389	10		1	0.991 (0.006)	0.982-0.996	0.941 (0.006)	0.932-0.946
Mean	10	2843	62		0.999	0.953 (0.011)	0.945-0.977	0.904 (0.011)	0.895-0.931

^a^AUROC: area under the receiver operating characteristic curve.

^b^WSS@100: work saved over sampling at 100%.

^c^WSS@95: work saved over sampling at 95%.

## Discussion

### Principal Results

These findings demonstrated that an active machine learning system could dramatically reduce the workload for performing systematic reviews of randomized controlled trials in several medical fields. Our data suggest that an active machine learning system could improve the precision of the systematic review process as well as reduce the time required, thus assisting with the development of clinical guidelines. In this study, the deep neural network–based active machine learning system achieved a 10-fold reduction in the literature screening workload for systematic reviews after a researcher initiated the learning process by randomly selecting 2 studies.

### Strengths and Limitations

We demonstrated that a 90% reduction in the workload for searching literature compared with manual assessment could be achieved and, whereas previous research mainly focused on small databases, we showed that this reduction in workload could be applied to large data sets by using systematic reviews of clinical studies. In addition, we specifically described the methods employed by our active machine learning system for systematic reviews of literature, which most previous reports do not explain.

One of the limitations of our study was the absence of a criterion for when active learning can be stopped. The study focused on how much workload could be reduced by the embedding-based technique using WSS@95%; however, active learning could increase the AUROC value as active learning steps proceeded; and therefore, at some point, this method could separate correct articles from incorrect articles in the learning process. The other limitation of the study was that 2 correct articles were required at the beginning of active learning. In practice, it may be challenging to start the review process with 2 correct articles already identified. This limitation might be overcome by using 2 consecutive systematic reviews on the same topic; the papers used in the first review could be used as the learning data to identify new articles for the second systematic review.

### Comparison With Prior Work

Several studies using text mining or computational technique to reduce workload in systematic reviews have been reported. Marshall et al [35] used an ensemble model consisting of support vector classification and convolutional neural networks to classify randomized controlled trial papers and showed that the model predicted randomized controlled trial papers (AUROC 0.987, 95% CI 0.984-0.989) and also discussed the automating risk of bias assessment using large corpus labeled by distant supervision and presented a step toward automating or semiautomating the data extraction needed for the synthesis of clinical trials [36]. These authors also evaluated the performance of the RobotReviewer in another paper [37] and showed that machine learning could help reviewers to detect sentences or documents containing risk of bias but are not be able to replace manual review by humans yet. However, these works showed a great potency of workload reduction in systematic reviews with machine learning techniques. Wallace et al [38] developed a tool for systematic review called Abstrackr. Based on its technical report [38], 2 case studies were tested, and a 40% workload reduction with 100% recall was achieved. Rathbone et al [39] evaluated the performance of Abstrackr for 4 systematic reviews and summarized that reduction of workload varied from 10% to 80%, but that precision was also decreased. Recently, Gates et al [40] evaluated the Abstrackr performance retrospectively against human review for 4 studies and concluded that it could reduce workload by 9.5% to 88.4%, varying by the screening task. A review of systematic reviews [41] noted that current use of text mining in systematic reviews could reduce workload from 30% to 70%, at 95% recall. As for other techniques to reduce workload in systematic reviews, using 17 studies, RobotAnalyst [42] was reported as an active learning approach using latent Dirichlet allocation to reduce workload, for which WSS@95% varied between 6.89% to 70.74%. Workload reduction varies by study or task; therefore, direct comparison with our study is difficult. However, our method, using an embedding-based technique, showed good performance with the 8 systematic review data sets of randomized controlled trials.

Regarding other embedding methods, embedding vectors from BioBERT-Base version 1.1 (4.5 billion PubMed abstracts, trained for 1 million steps) [43] were applied to the same 8 studies. WSS@95% was calculated for each study using the same algorithm. The mean WSS@95% for the 8 studies was 0.747 (SD 0.119), which was about 15% lower than the 0.904 (SD 0.02) from this study (Table 1). Fine-tuning for each study was not performed because some of the studies include only a small number of articles. Hence, the performance of BioBERT could be improved by fine-tuning. However, the method in the present paper is still competitive enough considering the performance and simplicity of the model.

We assessed systematic reviews and meta-analyses of randomized controlled trials because these can estimate the true efficacy and risks of treatment. In contrast, estimates derived from systematic reviews and meta-analyses of epidemiological studies are more limited due to the observational design of the underlying studies. Therefore, further investigation will be needed to assess the effectiveness of our system for meta-analyses of epidemiological studies. Furthermore, in the future, we plan to evaluate Cochrane review papers, which have a standardized review process.

### Conclusion

The deep neural network–based active machine learning system investigated in this study achieved at least a 10-fold reduction of the literature screening workload for systematic reviews after a researcher initiated the learning process by randomly selecting 2 studies that fulfilled the inclusion criteria for the target review. Our findings suggest that machine learning could facilitate the acquisition of evidence for developing new clinical guidelines.

## Appendix

Multimedia Appendix 1 Online supplementary material.

## Abbreviations

AUROC: area under the receiver operating characteristic curve
WSS: work saved over sampling
