# Supplementary material for: Deep Neural Network for Reducing the Screening Workload in Systematic Reviews for Clinical Guidelines: Algorithm Validation Study
Source: J Med Internet Res. 2020 Dec 30;22(12):e22422. doi: 10.2196/22422 (PMC7806440; doi:10.2196/22422)
Supplement: Multimedia Appendix 1 [file jmir_v22i12e22422_app1.docx]

**Online Supplementary material**

**Supplementary material 1**. Search strategies for reproducing the primary screening datasets

**Supplementary material 2**. Work saved over sampling

**Supplementary material 3**. Characteristics of the systematic reviews and meta-analyses

**Supplementary material 4.** Reproduction of the primary screening dataset for analysis

**Supplementary material 1**. **Search strategies** **for reproducing the primary screening datasets**

We reproduced the primary screening dataset, including abstracts, according to the search strategy described in each systematic review/meta-analysis.

1) Based on the search strategy for Ovid MEDLINE described in each systematic review/meta-analysis, we created a search strategy for PubMed. If the Ovid MEDLINE search used a method that was not available in PubMed (e.g., approximate operator), we employed another method that identified all of the articles assessed in the systematic review/meta-analysis.

2) PubMed was searched and the results were downloaded as a “MEDLINE” text file.

3) If it was difficult to reproduce the search strategy using PubMed, we created a search strategy for ProQuest MEDLINE, conducted a search, and downloaded the results. In this case, the download format was “Endnote, Reference manager style, [Brief citation/Abstract]” (text file).

4) If a search strategy for PubMed was described in a systematic review/meta-analysis, we employed the same strategy for our PubMed search.

5) We did not alter the search strategy described by the authors, even if an error was detected. We only revised it if the database could not handle a description.

6) We labeled the articles identified by the search strategy that were actually used in each systematic review/meta-analysis as “correct” articles and the articles that were not used as “incorrect” articles.

The search strategies used to reproduce the primary screening dataset (including abstracts) based on the strategy described in each review are set out below.

**1. Chatterjee S, et al. New oral anticoagulants and the risk of intracranial hemorrhage: traditional and Bayesian meta-analysis and mixed treatment comparison of randomized trials of new oral anticoagulants in atrial fibrillation. JAMA Neurol. 2013;70:1486–1490.**

PubMed

"randomized controlled trial"[pt]

random*[tiab] or placebo*[tiab] or single blind*[tiab] or double-blind*[tiab] or triple blind*[tiab]

retraction of publication[pt] or retracted publication[pt]

#1 or #2 or #3

animals[mh] not humans[mh]

(comment[pt] or editorial[pt] or meta-analysis[pt] or practice guideline[pt] or review[pt] or letter[pt] or journal correspondence[pt]) not "randomized controlled trial"[pt]

(random sampl*[tiab] or random digit*[tiab] or random effect*[tiab] or random survey[tiab] or random regression[tiab]) not "randomized controlled trial"[pt]

(oral[tiab] or direct[tiab]) and thrombin inhibitor*[tiab]

((factor*[tiab] or antifactor*[tiab]) and Xa inhibitor*[tiab]) or (factor drug*[tiab] or antifactor drug*[tiab])

apixaban[tiab] or eliquis[tiab]

BMS-562247-01[all]

dabigatran[tiab] or pradaxa[tiab] or pradax[tiab] or prazaxa[tiab]

EC3-4-21-5[all]

rivaroxaban[tiab] or xarelto[tiab]

BAY59-7939[all]

#4 not (#5 or #6 or #7)

#8 or #9 or #10 or #11 or #12 or #13 or #14 or #15

#16 and #17

#18 and 1946:2012[dp]

Data Sources and Searches

The following search algorithm was used for MEDLINE:

1. “randomized controlled trial”.pt.

2. (random$ or placebo$ or single blind$ or double-blind$ or

triple blind$).ti,ab.

3. (retraction of publication or retracted publication).pt.

4. or/1-3

5. (animals not humans).sh.

6. ((comment or editorial or meta-analysis or practiceguideline

or review or letter or journal correspondence) not

“randomized controlled trial”).pt.

7. (random sampl$ or random digit$ or random effect$ or random

survey or random regression).ti,ab. not “randomized

controlled trial”.pt.

8. ((oral or direct) adj3 thrombin inhibitor$).ti,ab.

9. ((factor$ or antifactor$) adj3 (Xa inhibitor$ or drug$)).ti,ab.

10. (apixaban or eliquis).ti,ab.

11. BMS-562247-01.mp.

12. (dabigatran or pradaxa or pradax or prazaxa).ti,ab.

13. EC3-4-21-5.mp.

14. (rivaroxaban or xarelto).ti,ab.

15. BAY59-7939.mp.)

It was modified and adapted for search of the CENTRAL,

CINAHL, and EBSCO databases and supplemented with

searches in conference abstract books and on http://www

.clinicaltrials.gov. The searcheswere performed up toDecember

1, 2012,with no language restriction. Reference lists of appropriate

review articles and of the original retrieved studies

were searched to identify studies potentially missed by the database

searches (eFigure 1 in Supplement).

**2. Apolonia García-Patterson, et al. Glibenclamide, metformin, and insulin for the treatment of gestational diabetes: a systematic review and meta-analysis. BMJ 2015;350:h102.**

PubMed

"Glyburide"[MeSH]

"Metformin"[MeSH]

oral diabetes agent*[tiab]

oral antidiabetic agent*[tiab]

oral hypoglycemic agent*[tiab]

metformin[tiab]

glyburide[tiab]

glibenclamide[tiab]

glimepiride[tiab]

glipizide[tiab]

sulfonylurea[tiab]

sulphonylurea[tiab]

(((((((((((#1) OR #2) OR #3) OR #4) OR #5) OR #6) OR #7) OR #8) OR #9) OR #10) OR #11) OR #12

"Pregnancy"[MeSH]

"Diabetes, Gestational"[MeSH]

pregnan*[tiab]

gestation*[tiab]

GDM[tiab]

((((#14) OR #15) OR #16) OR #17) OR #18

(#13) AND #19

(randomized controlled trial[pt] OR controlled clinical trial[pt] OR randomized[tiab] OR placebo[tiab] OR drug therapy[sh] OR randomly[tiab] OR trial[tiab] OR groups[tiab]) NOT (animals[mh] NOT (humans[mh] AND animals[mh]))

(#20) AND #21

#22 and 1946:2014/05/31[dp]

Supplementary table 1. Search strategy

MEDLINE (PubMed)

#1 “Glyburide”[MeSH] 5415

#2 "Metformin"[MeSH] 6142

#3 oral diabetes agent*[tiab] 12

#4 oral antidiabetic agent*[tiab] 658

#5 oral hypoglycemic agent*[tiab] 1406

#6 metformin[tiab] 8079

#7 glyburide[tiab] 1212

#8 glibenclamide[tiab] 6634

#9 glimepiride[tiab] 744

#10 glipizide[tiab] 830

#11 sulfonylurea[tiab] 4029

#12 sulphonylurea[tiab] 1331

#13 (((((((((((#1) OR #2) OR #3) OR #4) OR #5) OR #6) OR #7) OR #8) OR #9) OR #10) OR #11) OR #12 22521

#14 “Pregnancy”[MeSH] 682623

#15 “Diabetes, Gestational”[MeSH] 6200

#16 pregnan*[tiab] 353829

#17 gestation*[tiab] 138524

#18 GDM[tiab] 2545

#19 ((((#14) OR #15) OR #16) OR #17) OR #18 797198

#20 (#13) AND #19 909

#21 (randomized controlled trial[pt] OR controlled clinical trial[pt] OR randomized[tiab] OR placebo[tiab] OR drug

therapy[sh] OR randomly[tiab] OR trial[tiab] OR groups[tiab]) NOT (animals[mh] NOT (humans[mh] AND

animals[mh])) 2713046

#22 (#20) AND #21 567

3. **Mudaliar U, et al. Cardiometabolic risk factor changes observed in diabetes prevention programs in US settings: a systematic review and meta-analysis. PLoSMed 2016;13:e1002095**

PubMed

"Overweight/prevention and control"[Mesh]

"Obesity/prevention and control"[Mesh]

"Glucose Intolerance"[Mesh]

"Prediabetic State"[Mesh]

"Diabetes Mellitus/prevention and control" [Mesh]

"Diabetes Mellitus, Type 2/prevention and control"[Mesh]

"Prediabetic State/prevention and control"[Mesh]

"Metabolic Syndrome/prevention and control"[Mesh]

"Prediabetic State/therapy"[Mesh]

"Diabetes Prevention"[all]

"Diabetes risk reduction"[all]

#1 or #2 or #3 or #4 or #5 or #6 or #7 or #8 or #9 or #10 or #11

Weight Loss[all]

Lifestyle[all]

Preventive Health Services[all]

Program evaluation[all]

#13 or #14 or #15 or #16

#12 and #17

#18 and english[la]

#19 and humans[mh]

#20 and 2011/04/01:2016/05/01[dp]

Medline via PubMed (April 2011 to May 2016)

4,653 studies

#1 "Overweight/prevention & control"[Mesh]

#2 "Obesity/prevention & control"[Mesh]

#3 "Glucose Intolerance"[Mesh]

#4 "Prediabetic State"[Mesh]

#5 "Diabetes Mellitus/prevention & control" [Mesh]

#6 "Diabetes Mellitus, Type 2/prevention & control"[Mesh]

#7 "Prediabetic State/prevention & control"[Mesh]

#8 "Metabolic Syndrome X/prevention & control"[Mesh]

#9 "Prediabetic State/therapy"[Mesh]

#10 "Diabetes Prevention"

#11 "Diabetes risk reduction"

#12 OR / 1 -11

#13 Weight Loss

#14 Lifestyle

#15 Preventive Health Services

#16 Program evaluation

#17 OR/ 13-10

#18 12 AND 18

Limits: English, Humans, Publication Date from 2011/04/01 to 2013/04/01

**4. Yanovski SZ, Yanovski JA. Long-term drug treatment for obesity: a systematic and clinical review. JAMA 2014;311:74–86.**

PubMed

Obesity[mh] or obesity[tiab]

Appetite[mh] or appetite[tiab]

Satiety Response[mesh:noexp] or satiety[tiab]

#1 or #2 or #3

Drug Therapy[mh]

drug therapy[sh]

drug[tiab] or drugs[tiab]

pharmacotherap*[tiab]

orlistat[tiab]

phentermine[tiab]

diethylpropion[tiab]

phendimetrazine[tiab]

benzphetamine[tiab]

topiramate[tiab]

Qsymia[tiab]

Qnexa[tiab]

lorcaserin[tiab]

Belviq[tiab]

#5 or #6 or #7 or #8 or #9 or #10 or #11 or #12 or #13 or #14 or #15 or #16 or #17 or #18

Clinical Trials as Topic[mh]

Clinical Trial*[pt]

Controlled Clinical Trial[pt]

Randomized Controlled Trial[pt]

"clinical trial"[tiab] or "clinical trials"[tiab] or "clinical study"[tiab] or "clinical studies"[tiab]

(controlled[tiab] or randomized[tiab] or randomised[tiab]) and (trial[tiab] or trials[tiab] or study[tiab] or studies[tiab])

Meta-Analysis as Topic[mh]

Meta-Analysis[pt]

metaanaly*[tiab] or meta analy*[tiab]

#20 or #21 or #22 or #23 or #24 or #25 or #26 or #27 or #28

#4 and #19 and #29

#30 and 1946:2013/09/30[dp]

**5. Eng C, Kramer CK, Zinman B, Retnakaran R. Glucagon-like peptide-1 receptor agonist and basal insulin combination treatment for the management of type 2 diabetes: a systematic review and meta-analysis. Lancet 2014;384:2228–2234.**

PUbMed

insulins[mh] OR insulin[tiab] OR insulins[tiab] OR NPH[tiab] OR glargine[tiab] OR detemir[tiab] OR degludec[tiab]

glucagon-like peptide 1 receptor agonist[tiab] OR exenatide[tiab] OR liraglutide[tiab] OR lixisenatide[tiab] OR dulaglutide[tiab] OR albiglutide[tiab] OR semaglutide[tiab] OR taspoglutide[tiab]

#1 and #2

#3 and 1950:2014/07/31[dp]

Search strategy and selection criteria

This systematic review and meta-analysis is reported in

accordance with the Preferred Reporting Items for

Systematic Reviews and Meta-Analyses (PRISMA)

Statement and was registered at International

Prospective Register of Systematic Reviews (number

CRD42014010688).10

We selected relevant studies published between

Jan 1, 1950, and July 29, 2014, by searching Embase,

PubMed, Cochrane, Web of Knowledge, FDA.gov, and

ClinicalTrials.gov. We applied no language restrictions.

We used the following combined text and MeSH terms:

“insulin” and “glucagon-like peptide 1 receptor agonist”.

The complete search used for PubMed was: (insulin

[MeSH Terms] OR insulin [Text Word] OR NPH [Text

word] OR glargine [Text word] OR detemir [Text word]

OR degludec [Text word]) AND (glucagon-like peptide 1

receptor agonist [Text Word] OR exenatide [Text word]

OR liraglutide [Text word] OR lixisenatide [Text word] OR

dulaglutide [Text word] OR albiglutide [Text word]

OR semaglutide [Text word] OR taspoglutide (Text

word])). We considered all potentially eligible studies for

review, irrespective of the primary outcome or language.

We also did a manual search, using the reference lists of

key articles published in English.

6. **McBrien K, Rabi DM, Campbell N, et al. Intensive and standard blood pressure targets in patients with type 2 diabetes mellitus: systematic review and meta-analysis. Arch Intern Med 2012;172:1296–1303.**

PubMed

Diabetes Mellitus[mh]

diabet*[tiab]

#1 or #2

Hypertension[mh]

hypertens*[tiab]

Blood Pressure[mh]

blood pressure[tiab] or bloodpressure[tiab] or BP[tiab]

Antihypertensive Agents[mh]

antihypert*[tiab]

#4 or #5 or #6 or #7 or #8 or #9

#3 and #10

randomized controlled trial[pt]

controlled clinical trial[pt]

randomized[tiab]

placebo[tiab]

drug therapy[sh]

randomly[tiab]

trial[tiab]

groups[tiab]

#12 or #13 or #14 or #15 or #16 or #17 or #18 or #19

Animals[mh]

Humans[mh]

#21 not #22

#20 not #23

#11 and #24

(strict*[tiab] or tight*[tiab] or target*[tiab] or value[tiab] or values[tiab] or intens*[tiab] or below[tiab] or lower*[tiab]) and (blood pressure[tiab] or bloodpressure[tiab] or BP[tiab] or pressure[tiab] or pressures[tiab])

#25 and #26

#27 and 1948:2011/03/31[dp]

eAppendix 2 eTable 1. Medline search strategy

1. exp Diabetes Mellitus/

2. diabet$.tw.

3. 1 or 2

4. exp Hypertension/

5. hypertens$.tw.

6. exp Blood Pressure/

7. (blood pressure or bloodpressure or BP).tw.

8. exp Antihypertensive Agents/

9. antihypert$.tw.

10. 4 or 5 or 6 or 7 or 8 or 9

11. 3 and 10

12. randomized controlled trial.pt.

13. controlled clinical trial.pt.

14. randomized.ab.

15. placebo.ab.

16. drug therapy.fs.

17. randomly.ab.

18. trial.ab.

19. groups.ab.

20. 12 or 13 or 14 or 15 or 16 or 17 or 18 or 19

21. Animals/

22. Humans/

23. 21 not 22

24. 20 not 23

25. 11 and 24

26. ((strict$ or tight$ or target$ or value? or intens$ or below or

lower$) adj4 (blood pressure or bloodpressure or BP or

pressure?)).tw.

27. 25 and 26

7. **Andrade-Castellanos CA, Colunga-Lozano LE, Delgado-Figueroa N, Gonzalez-Padilla DA. Subcutaneous rapid-acting insulin analogues for diabetic ketoacidosis. Cochrane Database Syst Rev 2016;1:CD011281.**

PubMed

Diabetic Ketoacidosis[mesh:noexp]

Diabetic Coma[mesh:noexp]

hyperglycemic emergenc*[tiab] or diabetic emergenc*[tiab]

diabet*[tiab] and (keto*[tiab] or acidos*[tiab] or coma[tiab])

DKA[tiab]

#1 or #2 or #3 or #4 or #5

Insulin Lispro[mesh:noexp]

Insulin Aspart[mesh:noexp]

Insulin, Short-Acting[mesh:noexp]

glulisine[tiab] or apidra[tiab]

humulin[tiab] or novolin[tiab]

lispro[tiab] or aspart[tiab]

novolog[tiab] or novorapid[tiab]

insulin*[tiab] and analogue*[tiab]

acting insulin*[tiab]

#7 or #8 or #9 or #10 or #11 or #12 or #13 or #14 or #15

#6 and #16

animals[mh] not humans[mh]

#17 not #18

#19 and 1946:2015/10/31[dp]

PubMed

(hyperglycemic emergenc*[tw] OR hyperglycaemic emergenc*[tw] OR diabetic emergenc*[tw] OR (diabet*[tw] AND (ketoac*[tw] OR acidos*[tw] OR coma[tw])) OR DKA[tw])

(glulisine[tw] OR apidra[tw] OR humulin[tw] OR novolin[tw] OR lispro[tw] OR aspart[tw] OR novolog[tw] OR novorapid[tw] OR (insulin[tw] AND analog*[tw]) OR acting insulin*[tw])

#1 AND #2

#3 NOT medline[sb] NOT pmcbook

#4 and 1946:2015/10/31[dp]

MEDLINE (Ovid SP)

1. Diabetic Ketoacidosis/

2. Diabetic Coma/

3. ((hyperglyc?emic or diabet*) adj emergenc*).tw.

4. (diabet* and (keto* or acidos* or coma)).tw.

5. DKA.tw.

6. or/1-5

7. Insulin Lispro/

8. Insulin Aspart/

9. Insulin, Short-Acting/

10. (glulisine or apidra).tw.

11. (humulin or novolin).tw.

12. (lispro or aspart).tw.

13. (novolog or novorapid).tw.

14. (insulin* adj3 analogue*).tw.

15. acting insulin*.tw.

16. or/7-15

17. 6 and 16

18. exp animals/ not humans/

19. 17 not 18

PubMed

#1 (hyperglycemic emergenc*[tw]ORhyperglycaemic emergenc*[tw]ORdiabetic emergenc*[tw]OR(diabet*[tw]AND(ketoac*[tw]

OR acidos*[tw] OR coma[tw])) OR DKA[tw])

#2 (glulisine[tw] OR apidra[tw] OR humulin[tw] OR novolin[tw] OR lispro[tw] OR aspart[tw] OR novolog[tw] OR novorapid[tw]

OR (insulin[tw] AND analog*[tw]) OR acting insulin*[tw])

#3 #1 AND #2

#4 #3 NOT medline[sb] NOT pmcbook

8. **Arguedas JA, Leiva V, Wright JM. Blood pressure targets for hypertension in people with diabetes mellitus. Cochrane Database Syst Rev 2013;10:CD008277.**

PubMed

diabetes mellitus[mesh]

diabet*[tiab]

#1 OR #2

hypertension[mesh]

hypertens*[tiab]

blood pressure[mesh]

blood pressure[tiab] OR bloodpressure[tiab]

#4 OR #5 OR #6 OR #7

(strict*[tiab] OR target*[tiab] OR tight*[tiab] OR intens*[tiab] OR below[tiab]) AND (blood pressure[tiab] OR systolic[tiab] OR diastolic[tiab] OR bp[tiab] OR level*[tiab])

(bp[tiab] OR blood pressure[tiab]) AND lowering[tiab]

#9 OR #10

randomized controlled trial[pt]

controlled clinical trial[pt]

randomized[tiab]

placebo[tiab]

clinical trials as topic[mesh:noexp]

randomly[tiab]

trial[ti]

#12 OR #13 OR #14 OR #15 OR #16 OR #17 OR #18

animals[mesh:noexp] NOT (humans[mesh:noexp] AND animals[mesh:noexp])

#19 NOT #20

#3 AND #8 AND #11 AND #21

Appendix 1. MEDLINE search strategy

Database: Ovid MEDLINE(R) 1946 to Present with Daily Update

Search Date: 4 October 2013

--------------------------------------------------------------------------------

1 exp diabetes mellitus/

2 diabet$.tw.

3 or/1-2

4 exp hypertension/

5 hypertens$.tw.

6 exp blood pressure/

7 (blood pressure or bloodpressure).tw.

8 or/4-7

9 ((strict$ or target$ or tight$ or intens$ or below) adj3 (blood pressure or systolic or diastolic or bp or level$)).tw.

10 ((bp or blood pressure) adj2 lowering).tw.

11 or/9-10

12 randomized controlled trial.pt.

13 controlled clinical trial.pt.

14 randomized.ab.

15 placebo.ab.

16 clinical trials as topic/

17 randomly.ab.

18 trial.ti.

19 or/12-18

20 animals/ not (humans/ and animals/)

21 19 not 20

22 3 and 8 and 11 and 21

**Supplementary material 2.** **Work saved over sampling**

Work saved over sampling (WSS) was calculated as follows:

$$WSS@R=\frac{TN+FN}{N}-1.0+\frac{\mathrm{TP}}{TP+FN} =\frac{TN+FN}{N}-\left( 1.0-R \right)=\frac{TN+FN-(TP+FN+FP+TN)}{N}+R=R-\frac{TP+FP}{N}$$

where TP, FN, FP, TN, and R is the true positive rate, false negative rate, false positive rate, true negative rate, and recall rate, respectively. WSS at 95% recall (WSS@95%) was calculated by using the TN and FN values corresponding to a 95% recall rate. If both sides of the last equation are multiplied by N, N*0.95 gives the average number of articles that have to be reviewed to achieve a 95% recall rate when articles are selected manually. On the other hand, TP+FP=N*(TP+FP)/N gives the number of articles reviewed to achieve a 95% recall rate using Concept Encoder. Therefore, N*0.95-(TP+FP) gives the difference in the number of articles reviewed between machine learning and manual selection, so WSS@R corresponds to the reduction of the literature screening workload (number of articles reviewed) compared with manual selection. Similarly, WSS@100% corresponds to the reduction in the number of articles reviewed to achieve a 100% recall rate by machine learning compared with manual selection.

**Supplementary material 3.** Characteristics of the systematic reviews and meta-analyses

| **Ref.** | **Year** | **Review/meta-analysis** | **Size of the dataset** | **No. of studies reviewed (correct articles)** |
| --- | --- | --- | --- | --- |
| 21 | 2013 | Chatterjee S, et al. New oral anticoagulants and the risk of intracranial hemorrhage: traditional and Bayesian meta-analysis and mixed treatment comparison of randomized trials of new oral anticoagulants in atrial fibrillation. JAMA Neurol. 2013;70:1486–1490. | 410 | 6 |
| 22 | 2015 | Apolonia García-Patterson, et al. Glibenclamide, metformin, and insulin for the treatment of gestational diabetes: a systematic review and meta-analysis. BMJ 2015;350:h102. | 560 | 12 |
| 23 | 2016 | Mudaliar U, et al. Cardiometabolic risk factor changes observed in diabetes prevention programs in US settings: a systematic review and meta-analysis. PLoSMed 2016;13:e1002095 | 5644 | 17 |
| 24 | 2014 | Yanovski SZ, Yanovski JA. Long-term drug treatment for obesity: a systematic and clinical review. JAMA 2014;311:74–86. | 6935 | 20 |
| 25 | 2014 | Eng C, Kramer CK, Zinman B, Retnakaran R. Glucagon-like peptide-1 receptor agonist and basal insulin combination treatment for the management of type 2 diabetes: a systematic review and meta-analysis. Lancet 2014;384:2228–2234. | 830 | 11 |
| 26 | 2012 | McBrien K, Rabi DM, Campbell N, et al. Intensive and standard blood pressure targets in patients with type 2 diabetes mellitus: systematic review and meta-analysis. Arch Intern Med 2012;172:1296–1303. | 5839 | 5 |
| 27 | 2016 | Andrade-Castellanos CA, Colunga-Lozano LE, Delgado-Figueroa N, Gonzalez-Padilla DA. Subcutaneous rapid-acting insulin analogues for diabetic ketoacidosis. Cochrane Database Syst Rev 2016;1:CD011281. | 138 | 5 |
| 28 | 2013 | Arguedas JA, Leiva V, Wright JM. Blood pressure targets for hypertension in people with diabetes mellitus. Cochrane Database Syst Rev 2013;10:CD008277. | 2389 | 5 |

**Supplementary material 4.** Reproduction of the primary screening dataset for each systematic review/meta-analysis

We reproduced the primary screening dataset, including abstracts, according to the search strategy described in each systematic review/meta-analysis.

1) Based on the search strategy for Ovid MEDLINE described in each systematic review/meta-analysis, we created a search strategy for PubMed. If the Ovid MEDLINE search used a method that was not available in PubMed (e.g., approximate operator), we employed another method that identified all of the articles assessed in the systematic review/meta-analysis.

2) PubMed was searched and the results were downloaded as a “MEDLINE” text file.

3) If it was difficult to reproduce the search strategy using PubMed, we created a search strategy for ProQuest MEDLINE, conducted a search, and downloaded the results. In this case, the download format was “Endnote, Reference manager style, [Brief citation/Abstract]” (text file).

4) If a search strategy for PubMed was described in a systematic review/meta-analysis, we employed the same strategy for our PubMed search.

5) We did not alter the search strategy described by the authors, even if an error was detected. We only revised it if the database could not handle a description.

6) We labeled the articles identified by the search strategy that were actually used in each systematic review/meta-analysis as “correct” articles and the articles that were not used as “incorrect” articles.
